# Real-time Analysis of Skin Biopsy Specimens With 2-Photon Fluorescence Microscopy

**DOI:** 10.1001/jamadermatol.2022.3628

**Published:** 2022-09-07

**Authors:** Vincent D. Ching-Roa, Chi Z. Huang, Sherrif F. Ibrahim, Bruce R. Smoller, Michael G. Giacomelli

**Affiliations:** 1 Department of Biomedical Engineering, University of Rochester, Rochester, New York; 2 Department of Dermatology, University of Rochester Medical Center, Rochester, New York; 3 Rochester Dermatologic Surgery, PC, Victor, New York; 4 Department of Pathology and Laboratory Medicine, University of Rochester Medical Center, Rochester, New York

## Abstract

**Importance:**

Nonmelanoma skin cancers (NMSCs) are primarily diagnosed through paraffin section histologic analysis of skin biopsy specimens that requires days to weeks before a formal diagnosis is reported. Two-photon fluorescence microscopy (TPFM) has the potential for point-of-care diagnosis of NMSC and other dermatologic conditions, which could enable same-visit diagnosis and treatment.

**Objective:**

To demonstrate that TPFM imaging of NMSC can occur within minutes of obtaining biopsies and provide similar histological features to those of conventional histology and evaluate TPFM diagnostic performance with respect to conventional histology.

**Design, Setting, and Participants:**

This comparative effectiveness pilot study examined 29 freshly excised biopsies from confirmed NMSC lesions in patients presenting for treatment. Biopsies underwent imaging immediately with TPFM on site at Rochester Dermatologic Surgery (Victor, New York) between October 2019 and August 2021. The imaged biopsies were subsequently submitted for paraffin histology to produce coregistered images. Twelve of these coregistered image pairs (41.4%) were used as a training set. Fifteen (51.7%) were used in a masked evaluation by a board-certified dermatopathologist. Two (6.9%) were excluded from the study before evaluation because they could not be coregistered.

**Main Outcomes and Measures:**

Sensitivity, specificity, and accuracy of TPFM for NMSC biopsies were evaluated compared with conventional histology.

**Results:**

Fourteen of the 15 biopsy specimens (93.3%) in the evaluation set were identically diagnosed with TPFM and paraffin histology. The TPFM had 100% sensitivity (95% CI, 48%-100%), 100% specificity (95% CI, 69%-100%), and 100% accuracy (95% CI, 78%-100%) for basal cell carcinoma diagnosis. For squamous cell carcinoma diagnosis, TPFM had 89% sensitivity (95% CI, 52%-100), 100% specificity (95% CI, 54%-100%), and 93% accuracy (95% CI, 68%-100%). For overall NMSC diagnosis, TPFM had a 93% sensitivity (95% CI, 66%-100%), 100% specificity (95% CI, 3%-100%), and 93% accuracy (95% CI, 68%-100%). Examination of the 1 discordant pair revealed mismatched imaging planes as the source of error.

**Conclusions and Relevance:**

The results of this comparative effectiveness pilot study suggest that TPFM captures histological characteristics of NMSC that are present in conventional histology, which reveals its potential as a rapid, point-of-care diagnostic alternative that does not need extensive sample preparation or retraining for image evaluation. Further validation of TPFM imaging performed for a larger cohort is needed to fully evaluate its diagnostic accuracy and potential effect within the field.

## Key Points

**Question:**

Can 2-photon fluorescence microscopy enable rapid point-of-care diagnosis of nonmelanoma skin cancer through real-time imaging of unprocessed fresh tissue biopsies?

**Findings:**

In this pilot comparative effectiveness research study using 2-photon fluorescence microscopy on 15 biopsies of known non-melanoma skin cancer lesions, key histological characteristics present in conventional histology were similarly detected with 2-photon fluorescence. Diagnosis of basal cell carcinoma displayed perfect accuracy (100% sensitivity and specificity), while diagnosis of squamous cell carcinoma revealed high accuracy (89% sensitivity and 100% specificity).

**Meaning:**

The study results suggest that 2-photon fluorescence microscopy may facilitate real-time diagnosis of nonmelanoma skin cancers and other dermatologic conditions with high accuracy, potentially reducing the delays associated with conventional histologic processing.

## Teaser

This comparative effectiveness research study examines the ability of 2-photon fluorescence microscopy to capture characteristic nonmelanoma skin cancer features.

## Introduction

Nonmelanoma skin cancer (NSMC) is the most common type of human cancer, with more cases annually than all other types of cancer combined in the US.^1^ Estimates from Medicare databases suggest around 9600 newly diagnosed daily cases of NMSC,^1^ with approximately 80% being basal cell carcinoma (BCC) and 20% being squamous cell carcinoma (SCC). Skin biopsy remains the criterion standard for diagnosis, in which a portion of a suspected lesion is excised, fixed, paraffinized, stained, and mounted on slides before evaluation by a dermatopathologist. This process requires several days from the time of biopsy to diagnosis, resulting in a delay of treatment and additional clinic visits for definitive care. Previous work has shown that approximately 70% of patients with a diagnosis of NMSC prefer to have same-day biopsy and treatment to avoid incurred additional costs or inconveniences because of travel and missed work days.^2^ Same-day treatments are also associated with increased clinical throughput and reduced likelihood of wrong-site surgeries.^2^ While same-day treatments can be achieved with frozen sections,^3^ the approach has variable reliability (83% to 93% concordance rates)^3,4,5,6,7^ because of freezing and disruption artifacts, as well as its dependence on highly skilled technicians and dedicated frozen section laboratories for tissue preparation.

Recent advances in microscopy and imaging techniques have allowed for nondestructive optical sectioning that allows for detection and diagnosis of NMSC either through noninvasive in-vivo imaging or slide-free histology. Imaging technologies, such as optical coherence tomography,^8,9,10^ reflectance confocal microscopy,^11,12,13^ and 2-photon fluorescence microscopy (TPFM),^14,15,16^ have been investigated and commercialized for in vivo imaging of NMSC. While in vivo imaging techniques can provide noninvasive diagnostic information, they are label free and rely on intrinsic properties of tissues (eg, refractive index, absorptivity, thermoelasticity, and autofluorescence); thus, they do not directly visualize conventional histological features, such as nuclei and stroma. Consequently, these images reveal features dissimilar to that of traditional histology slides and require extensive retraining for interpretation. Additionally, in vivo imaging is usually limited in either imaging depth, field of view, and/or spatial resolution,^17^ making its adaption as an alternative to conventional histology difficult. While label-free, slide-free techniques exist, such as with photoacoustic remote sensing microscopy^18^ and optical coherence tomography,^19^ one of the main advantages of slide-free histology is the ability to introduce fluorescent stains into the tissue to label nuclei and/or stroma directly. The use of exogenous stains enables visualization of similar features to hematoxylin-eosin (H&E) and, when combined with computational techniques, can generate virtually stained histology images that closely resemble conventional histology.^20^ These include imaging with deep ultraviolet microscopy,^21,22^ confocal fluorescence microscopy,^23,24,25,26,27,28^ and TPFM.^29^ Studies of NMSC surgical margins with confocal fluorescence microscopy have shown that these techniques provide good agreement with conventional histology.^25,26,27^ Further studies of discarded, frozen Mohs specimens using DNA and stromal fluorescent stains combined with pink and purple virtual staining to resemble conventional H&E have shown even higher agreement while requiring only limited training for image interpretation.^24^

Similar to confocal fluorescence microscopy, TPFM can generate high-resolution, virtually H&E-stained images, but with the further advantage of using near-infrared light that penetrates deeper through tissue,^30,31^ making it advantageous for rapid imaging of fresh, irregularly shaped biopsies with minimal preparation. By comparison, confocal microscopy is limited to superficial imaging of the tissue surface and is more strongly obscured by debris, which may account for the relatively high rate of excluded samples in some confocal studies.^24^ Finally, TPFM with virtual staining can easily be performed at video rate, for multiple tissue specimens in parallel, and in real-time, enabling rapid diagnosis.

This study aims to investigate the ability of TPFM to capture characteristic NMSC features and evaluate TPFM for real-time NMSC diagnosis of fresh biopsy samples. Digital TPFM images of NMSC biopsies were acquired immediately after excision and assessed compared with their corresponding coregistered H&E slide images.

## Methods

### Biopsy Preparation, Imaging, and Processing

**Figure 1.**
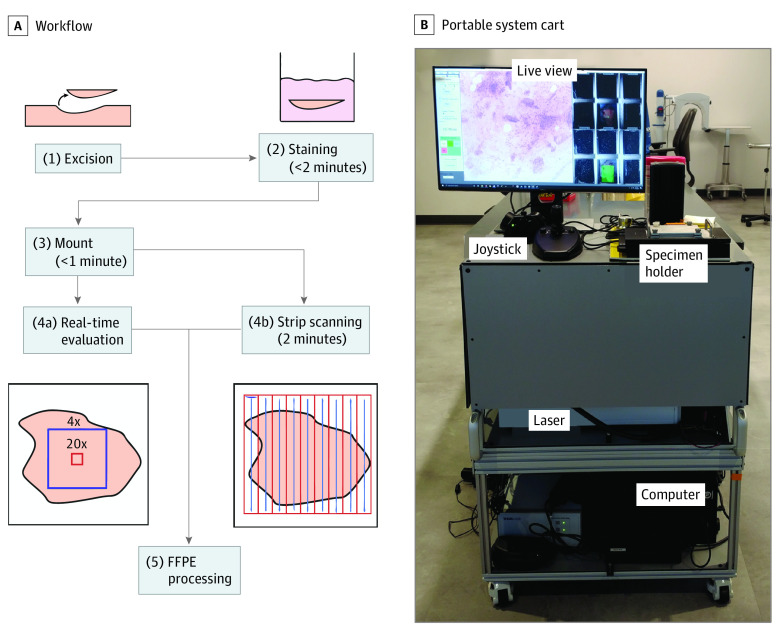
Clinical 2-Photon Fluorescence Microscopy (TPFM) Workflow and Portable System Cart A, Workflow for biopsy specimens used in this study. Excisions are ready to undergo imaging within 2 to 3 minutes. Real-time evaluation mode allows the 4× (blue box) and 20× field (red box) to be panned. Strip scanning acquires a full tissue mosaic in around 2 minutes. B, Clinic-based TPFM showing the physical user interface with live-view monitor and joystick for movement, while optical components, laser, and computer are enclosed in the cart.

Within an institutional review board protocol approved by the University of Rochester Medical Center (Rochester, New York), biopsy specimens from 29 patients with known NMSC were collected. Patients had confirmed NMSC from previous biopsies with clinically visible residual disease and were presenting for Mohs micrographic surgery. Biopsies were acquired by shave, curettage, and punch techniques. The workflow for tissue collection is summarized in Figure 1A. In all cases, freshly obtained biopsies were stained in a solution containing the DNA label acridine orange (A1301; Thermofisher) and the eosin-analogue sulforhodamine 101 (#80101; Biotium) at less than 1 mM concentration for 2 minutes. These agents rapidly permeate deep into tissue, labeling fresh specimens similar to H&E without the need for traditional staining, sectioning, freezing, or paraffinization.^32^ Whole biopsy specimens were then gently compressed with foam flat on their en face cut surface on a glass window and underwent imaging without additional preparation. Imaging the en face cut surface instead of the internal bread-loafed surface was performed to maximize surface area for better coregistration with subsequent paraffin sections. Imaging with TPFM can be performed in 2 ways: (1) real-time imaging, in which a user views video-rate virtually stained images on a computer monitor, similar to a standard microscope with variable objectives and manual translation, and (2) automated strip imaging, in which the system will rapidly image and record the specimen according to user-set volumetric bounds. For this study, all biopsy images were acquired with automated strip imaging for digital evaluation at a later point.

Immediately after imaging, formalin was introduced inside the sealed specimen holder for at least an hour to stiffen tissues, preserving their orientation and minimizing deformation of the specimens’ TPFM image plane. The partially fixed specimens were then removed from the specimen holder and stored in formalin for at least an additional day to ensure complete fixation before paraffin processing. Superficial sections were taken from each paraffin-embedded block and were stained with H&E as per standard procedure. This process enables cutting of paraffin sections that are relatively closely aligned with the original TPFM image plane. Finally, the H&E slides were scanned with an automated slide scanner (VS120; Olympus). Acridine orange and sulforhodamine 101 are completely removed during paraffin processing, particularly by xylene.^29^ Thus, this approach does not affect downstream histological processing or potential immunohistochemistry if needed.

### Clinical 2-Photon Fluorescence Microscope

The clinical TPFM uses a 1040-nm laser (YLMO; Menlo Systems) that scans across the specimen and excites fluorescence from acridine orange and sulforhodamine 101 at 16 frames per second. Emitted fluorescence from fluorophores is split and filtered into 518- to 558-nm and 620- to 680-nm bands to capture acridine orange and sulforhodamine 101, respectively. The 2 fluorescence channels are detected with 2 silicon photomultipliers that enable high-speed, high-signal-to-noise imaging while being robust to signals from possible contaminants, such as surgical ink.^33,34^ In addition, the microscope is self-contained in a light-tight enclosure, enabling imaging with room lights on. The microscope can alternate between a 4×, 0.28 numerical aperture air objective (XLFLUOR4X/340; Olympus) and a 20×, 0.7 numerical aperture air objective (UCPLFLN20X, Olympus), with 4 × 4 mm and 0.9 × 0.9 mm fields of view, respectively. Digital zooming is used to create a 2 × 2 mm 10 × field. Six specimen windows (25 mm × 30 mm) allow multiple biopsies to be mounted and imaged concurrently. Overall, the system achieves 16 frames per second with real-time imaging and up to 32 megapixels per second with automated strip imaging.^35^ The laser, microscope, controllers, and computer are all integrated and mounted onto a mobile cart, as shown in Figure 1B.

### Image Coregistration and Randomization

All 29 pairs of TPFM mosaics and corresponding H&E-stained permanent sections were assessed for image coregistration by the lead author (V.C.) before evaluation by a dermatopathologist (B.S.). A comprehensive list of the biopsies and the images is provided in the eTable in the Supplement. Two pairs of images were excluded from the study because the H&E and TPFM imaging planes were grossly different, precluding analyses of similar images. Of the remaining 27 coregistered image pairs, 2 were identified as having significant artifacts obscuring part of the paraffin section and 2 had portions of the specimens not visible in the TPFM images. These 4 specimens were placed in a training set used to familiarize the evaluator with the appearance of TPFM histology to avoid comparing diagnoses of incompletely coregistered images. From the remaining 23 samples, 15 were randomly selected for the evaluation set. The remaining 8 were added to the training set with the aforementioned 4, for a total of 12 samples in the training set.

### Study Design

To gain familiarity with the appearance of TPFM histology, 12 biopsies of the training set were reviewed as paired, side-by-side mosaics of coregistered TPFM and brightfield H&E images by a senior dermatopathologist (B.S.) who was not involved in sample selection or randomization and did not have previous experience evaluating TPFM images. After reviewing the biopsies in the training set, a link was provided to a customized web-based slide viewer that enables variable magnification in a way similar to Google Maps. The slide viewer included a form in which the diagnosis (benign, BCC, SCC, or other) could be recorded electronically. The viewer was configured to randomly display either the TPFM or permanent section image for each of the 15 biopsies in the evaluation set. After a 2-week delay, the second half of the set comprising the counterpart images was identically evaluated. Following evaluation, the diagnosis using TPFM was compared with the diagnosis from permanent sections.

## Results

### Similarity in BCC and SCC Features With TPFM and Paraffin H&E

**Figure 2.**
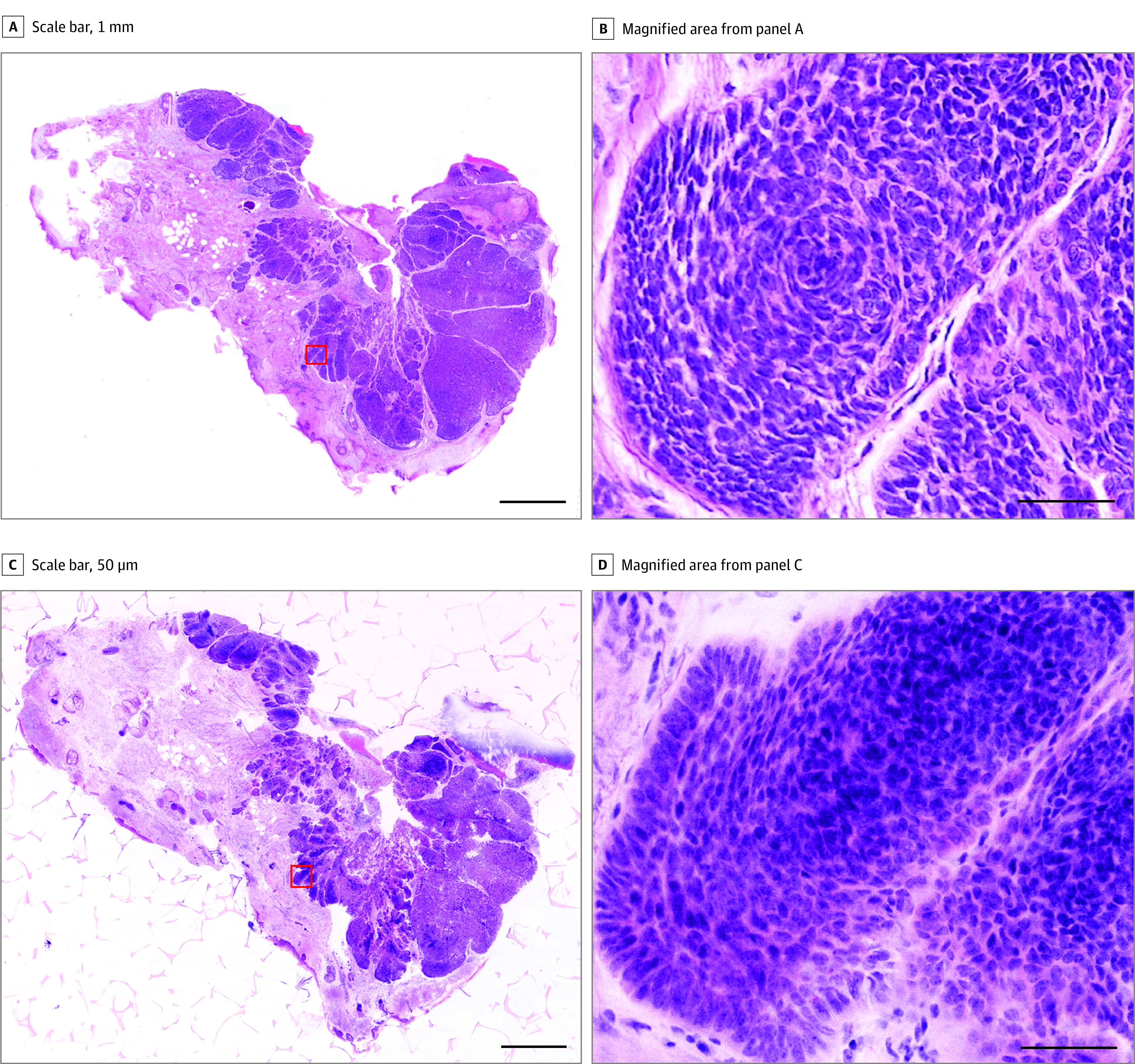
Nodular Basal Cell Carcinoma Full field brightfield image of a nodular basal cell carcinoma shave biopsy (A) and Magnified image of a region highlighted by the red box from panel A (B). The TPFM image of the same biopsy (C) and magnified region from panel C (D). (Scale bars: 1 mm [A and C], 50 μm [B and D]). Full H&E image: https://imstore.circ.rochester.edu/papers/jama2022/fig2/slide/zstack.html. Full TPFM image: https://imstore.circ.rochester.edu/papers/jama2022/fig2/tpfm/zstack.html

**Figure 3.**
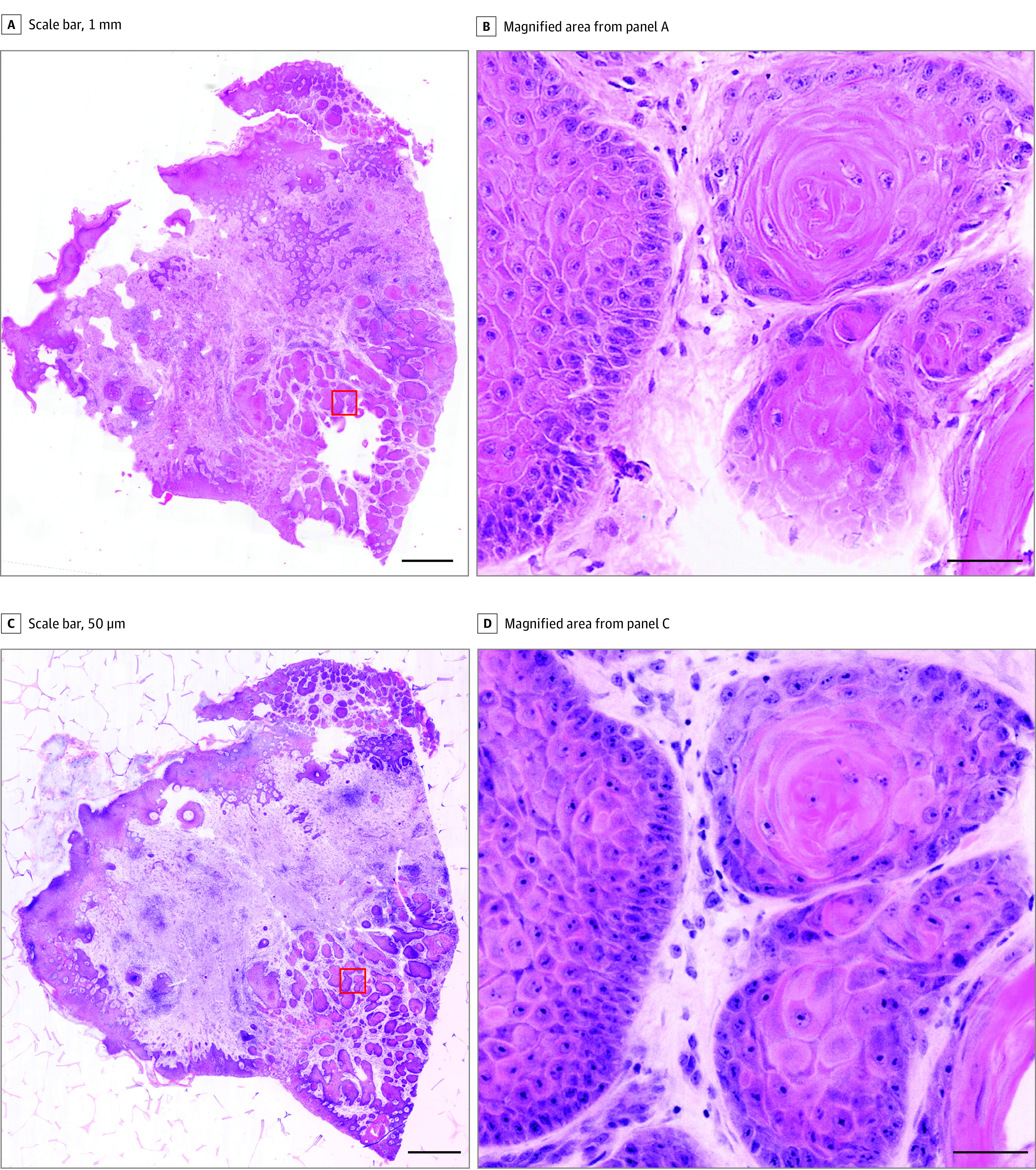
Squamous Cell Carcinoma Full field brightfield image of a squamous cell carcinoma shave biopsy (A) and magnified image of the red box from panel A (B). The TPFM image of the same biopsy (C) and magnified region from panel C (D). (Scale bars: 1 mm [A and C], 50 μm [B and D]). Full H&E image: https://imstore.circ.rochester.edu/papers/jama2022/fig3/slide/zstack.html. Full TPFM image: https://imstore.circ.rochester.edu/papers/jama2022/fig3/tpfm/zstack.html

The TPFM images of NMSC biopsies and their corresponding brightfield H&E showed excellent coregistration for BCC and SCC, as shown in Figure 2 and Figure 3. Basal cell carcinoma was recognized by islands of deep blue cells with a peripheral palisade within the dermis. The cells displayed a high nuclear to cytoplasmic ratio and were often seen coursing in a myxoid, cellular stroma. In some cases, follicular differentiation was seen. Central necrosis was variably present within the epithelial islands. Squamous cell carcinoma was recognized by tongues of eosinophilic epithelial islands present within the dermis. In some cases, these tongues extended down from the overlying epidermis, and in other areas, islands of keratinocytes were not connected. The cells demonstrated varying degrees of pleomorphism and cellular atypia. More poorly differentiated lesions demonstrated smaller islands and single atypical keratinocytes coursing between collagen bundles.

### Quantitative Evaluation of TPFM on NMSC

**Figure 4.**
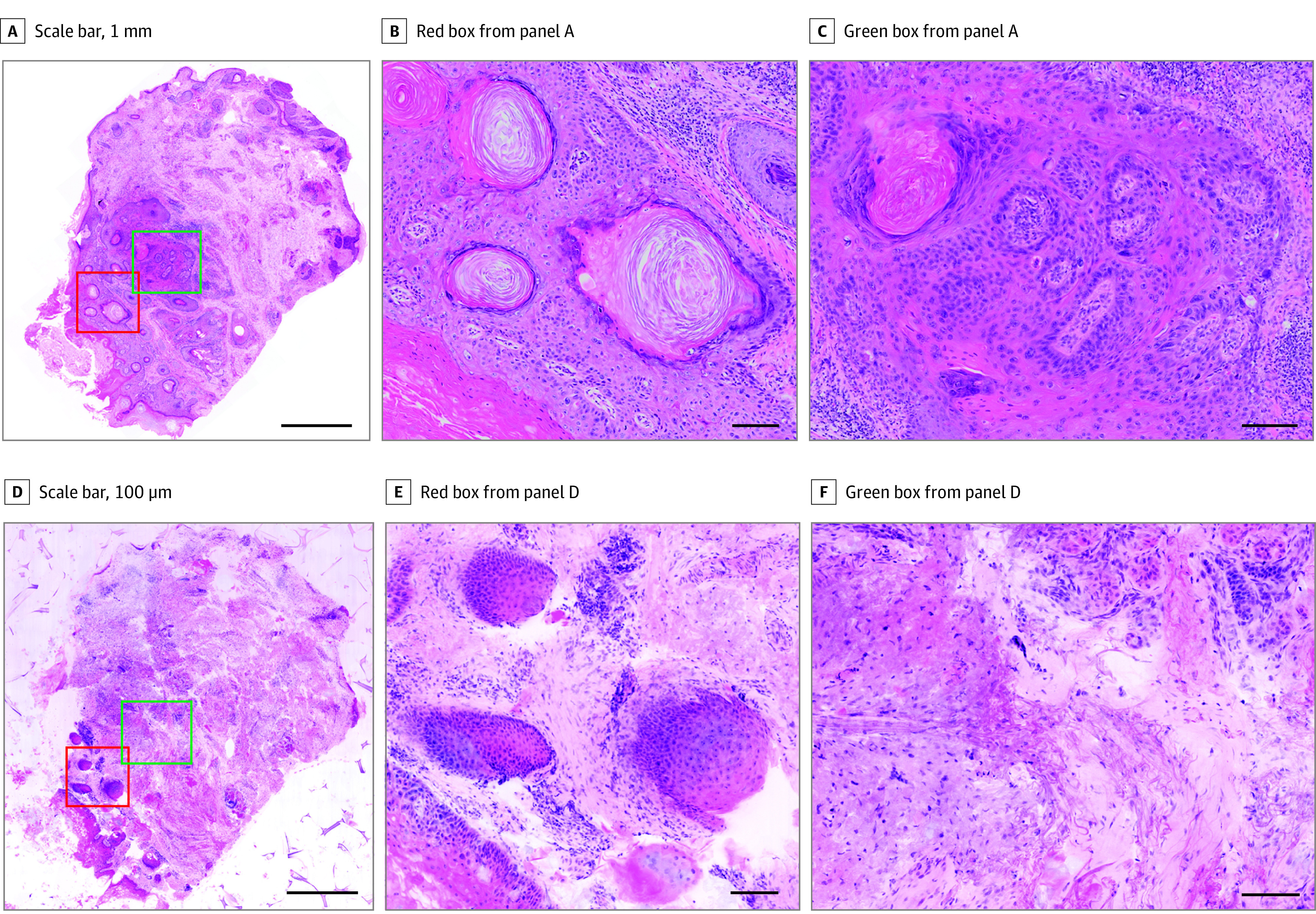
False Negative Squamous Cell Carcinoma Full field brightfield image of a squamous cell carcinoma shave biopsy (A) with magnified image of a region highlighted by the red box from panel A showing acellular keratinized pearls (B). C, Magnified image of a region highlighted by the green box from panel A showing degrees if dysplasia and keratinization. The TPFM image of the same shave biopsy (D) with magnified image of the same region highlighted in red (E). F, Magnified image of the same region highlighted in green. Both magnified TPFM images show absence of keratinization, suggesting different image plane depths or tilting of the paraffin section plane compared with the TPFM plane. (Scale bars: 1 mm [A and D], 100 μm [B,C, E, and F]). Full H&E image: https://imstore.circ.rochester.edu/papers/jama2022/fig4/slide/zstack.html. Full TPFM image: https://imstore.circ.rochester.edu/papers/jama2022/fig4/tpfm/zstack.html

Of the 15 biopsies, 5 (33.3%) were diagnosed positive for BCC, 9 (60.0%) were diagnosed positive for SCC, and 1 (6.7%) was diagnosed negative with H&E histology. With H&E histology as the standard, TPFM had a 93% sensitivity (95% CI, 66%-100%), 100% specificity (95% CI, 3%-100%), and 93% accuracy (95% CI, 68%-100%) when classifying between positive and negative diagnoses across all samples. The TPFM performed perfectly across all BCC samples while SCC demonstrated an 89% sensitivity (95% CI, 52%-100%) and 100% specificity (95% CI, 54%-100%). Results from the statistical analysis are summarized in the Table. Only 1 biopsy was diagnosed positive for SCC through H&E but negative with TPFM. This 1 false negative was because of a mismatch in imaging planes of both modalities, as shown in Figure 4. Examination of the zoomed in region in Figure 4, B and E, reveals exposed and clearly sectioned keratinized pearls, whereas only the surrounding cellular region without keratinization is captured with TPFM, suggesting that the paraffin section was cut substantially deeper compared with the imaging plane of the TPFM. This can be further seen in Figure 4, C and F, where again the brightfield H&E image shows an exposed keratinized pearl and some region of moderate cellular nuclear pleomorphism while the TPFM imaging plane only captures a more superficial, nondysplastic region.

**Table.** Statistics for TPFM BCC and SCC Diagnosis

Characteristic	(95% CI)		
	BCC	SCC	Total (n = 15)
Sensitivity	1.00 (0.48-1.00)	0.89 (0.52-1.00)	0.93 (0.66-1.00)
Specificity	1.00 (0.69-1.00)	1.00 (0.54-1.00)	1.00 (0.03-1.00)
PPV	1.00	1.00	1.00
NPV	1.00	0.86 (0.49-0.97)	0.50 (0.13-0.87)
Accuracy	1.00 (0.78-1.00)	0.93 (0.68-1.00)	0.93 (0.68-1.00)

Abbreviations: BCC, basal cell carcinoma; NMSC, nonmelanoma skin cancers; NPV, negative predictive value; PPV, positive predictive value; SCC, squamous cell carcinoma; TPFM, 2-photon fluorescence microscopy.

## Discussion

In this study, we reported the ability to histologically evaluate fresh tissue specimens markedly faster (within 2-3 minutes) than frozen or paraffin sections without the need for a histopathology laboratory or specialized personnel, making TPFM a potentially promising method for rapidly evaluating biopsies and other skin specimens, such as Mohs surgery stages. In contrast to other real-time techniques, such as reflectance confocal microscopy, TPFM enables H&E coloring of fluorescence images and video-rate operation that is similar to a conventional histology microscope, allowing for routine image interpretation. The prototype device is smaller than a cryotome, portable, and requires substantially less operator training than standard tissue processing. This allows for real-time, point-of-care interpretation of skin biopsies, even for low-resource settings. Furthermore, TPFM imaging is nondestructive and stains are removed by paraffinization^29^; thus TPFM imaging does not preclude subsequent histology or immunohistochemistry. A disadvantage of slide-free histology techniques in general is that histological evaluation is limited to the tissue surface. While TPFM enables deeper imaging than other fluorescent imaging techniques, imaging is still restricted to approximately 100 microns into tissue. However, as with conventional histology, specimens can be bisected or bread-loafed to expose internal tissue for imaging, eliminating the need for deeper imaging. Although we did not bisect or bread-loaf specimens to optimize coregistration with paraffin sections, in a clinical workflow, biopsies could be prepared and imaged in the same manner as conventional sections.

This comparative effectiveness pilot study shows potentially promising results for NMSC diagnosis, and while 1 discordant pair was present in the study, closer inspection revealed that the discrepancy was most likely because of a difference in image plane sampling and not because of an error in interpreting the TPFM image. Sampling errors can occur because paraffinization distorts tissue while permanent sections may be cut from different depth or angle compared with the TPFM image plane on fresh tissue. While the use of en face sections in this study facilitated more accurate coregistration, smaller bread-loaf could be used in a real-world setting where coregistration would not be necessary. In this study, prescreening of the TPFM and H&E image pairs solely for coregistration was performed by someone without a dermatopathology background to reduce sampling errors without introducing bias in the evaluation. In contrast to a previous study of surgical margins using confocal fluorescence microscopy in which nearly one-third of specimens were excluded for image quality or lack of coregistration,^24^ only 4 samples were rejected or used for training because of TPFM image quality or lack of registration, suggesting that the ability of TPFM to image deeper into specimens may be advantageous for imaging fresh biopsy tissues with irregular surfaces.

### Limitations

Limitations of this study involve having a small number of biopsies, limited number of possible diagnoses, and single dermatopathologist review. Furthermore, straightforward diagnoses without subtype analysis were performed. Future studies addressing these limitations and incorporating benign dermatologic conditions that are representative of typical clinic populations will be required to fully assess the ability of TPFM for immediate biopsy evaluation. Finally, future studies will be required to assess the use of TPFM in nonbiopsy workflows, such as Mohs surgery, for which comprehensive margin assessment is required.

## Conclusions

In this comparative effectiveness research study, TPFM reproduced histological characteristics of NMSC that are present in conventional histology and provided high concordance with paraffin histology on a masked evaluation of a small cohort. While these results suggest potential as a rapid, point-of-care diagnostic tool that requires no extensive sample preparation or retraining for image evaluation, further validation of TPFM imaging in a larger cohort is necessary to fully evaluate diagnostic accuracy.

## Notes

Supplement. eTable. List of collected skin biopsies
