# Supplementary material for: Real-time Analysis of Skin Biopsy Specimens With 2-Photon Fluorescence Microscopy
Source: JAMA Dermatol. 2022 Sep 7;158(10):1175–82. doi: 10.1001/jamadermatol.2022.3628 (PMC9453637; doi:10.1001/jamadermatol.2022.3628)
Supplement: Supplement. — eTable. List of collected skin biopsies [file jamadermatol-e223628-s001.pdf]

# Supplemental Online Content

Ching-Roa VD, Huang CZ, Ibrahim SF, Smoller BR, Giacomelli MG. Real-time analysis of skin biopsy specimes with 2-photon fluorescence microscopy. *JAMA Dermatol*. Published online September 7, 2022. doi:10.1001/jamadermatol.2022.3628

**eTable.** List of collected skin biopsies

This supplemental material has been provided by the authors to give readers additional information about their work.

© 2022 Ching-Roa VD et al. *JAMA Dermatology.*

**eTable. List of collected skin biopsies**

| ID | Set | Comments | | Image pair link |
|---|---|---|---|---|
| 1 | Training | | | https://imstore.circ.rochester.edu/papers/jama2022/esup1/compare2.html |
| 4 | Training | | | https://imstore.circ.rochester.edu/papers/jama2022/esup4/compare2.html |
| 5 | Training | | | https://imstore.circ.rochester.edu/papers/jama2022/esup5/compare2.html |
| 9 | Training* | Obscured coverage with TPFM | | https://imstore.circ.rochester.edu/papers/jama2022/esup9/compare2.html |
| 14 | Training | | | https://imstore.circ.rochester.edu/papers/jama2022/esup14/compare2.html |
| 16 | Training | | | https://imstore.circ.rochester.edu/papers/jama2022/esup16/compare2.html |
| 17 | Training* | Obscured coverage with H&E | | https://imstore.circ.rochester.edu/papers/jama2022/esup17/compare2.html |
| 18 | Training | | | https://imstore.circ.rochester.edu/papers/jama2022/esup18/compare2.html |
| 20 | Training | | | https://imstore.circ.rochester.edu/papers/jama2022/esup20/compare2.html |
| 22 | Training* | Obscured coverage with TPFM | | https://imstore.circ.rochester.edu/papers/jama2022/esup22/compare2.html |
| 23 | Training | | | https://imstore.circ.rochester.edu/papers/jama2022/esup23/compare2.html |
| 26 | Training* | Obscured coverage with H&E | | https://imstore.circ.rochester.edu/papers/jama2022/esup26/compare2.html |
| 8 | Excluded** | Grossly different imaging planes | | https://imstore.circ.rochester.edu/papers/jama2022/esup8/compare2.html |
| 24 | Excluded** | Grossly different imaging planes | | https://imstore.circ.rochester.edu/papers/jama2022/esup24/compare2.html |
| | | **TPFM diagnosis** | **H&E diagnosis** | |
| 2 | Evaluation | SCC | SCC | https://imstore.circ.rochester.edu/papers/jama2022/esup2/compare2.html |
| 3 | Evaluation | SCC | SCC | https://imstore.circ.rochester.edu/papers/jama2022/esup3/compare2.html |
| 6 | Evaluation | SCC | SCC | https://imstore.circ.rochester.edu/papers/jama2022/esup6/compare2.html |
| 7 | Evaluation | BCC | BCC | https://imstore.circ.rochester.edu/papers/jama2022/esup7/compare2.html |

© 2022 Ching-Roa VD et al. *JAMA Dermatology.*

| | | | | |
|---|---|---|---|---|
| 10 | Evaluation | SCC | SCC | https://imstore.circ.rochester.edu/papers/jama2022/esup10/compare2.html |
| 11 | Evaluation | BCC | BCC | https://imstore.circ.rochester.edu/papers/jama2022/esup11/compare2.html |
| 12 | Evaluation | BCC | BCC | https://imstore.circ.rochester.edu/papers/jama2022/esup12/compare2.html |
| 13 | Evaluation | BCC | BCC | https://imstore.circ.rochester.edu/papers/jama2022/esup13/compare2.html |
| 15 | Evaluation | SCC | SCC | https://imstore.circ.rochester.edu/papers/jama2022/esup15/compare2.html |
| 19 | Evaluation | BCC | BCC | https://imstore.circ.rochester.edu/papers/jama2022/esup19/compare2.html |
| 21 | Evaluation | Negative | SCC | https://imstore.circ.rochester.edu/papers/jama2022/esup21/compare2.html |
| 25 | Evaluation | Negative | Negative | https://imstore.circ.rochester.edu/papers/jama2022/esup25/compare2.html |
| 27 | Evaluation | SCC | SCC | https://imstore.circ.rochester.edu/papers/jama2022/esup27/compare2.html |
| 28 | Evaluation | SCC | SCC | https://imstore.circ.rochester.edu/papers/jama2022/esup28/compare2.html |
| 29 | Evaluation | SCC | SCC | https://imstore.circ.rochester.edu/papers/jama2022/esup29/compare2.html |

© 2022 Ching-Roa VD et al. *JAMA Dermatology.*
